# Discordance of immunohistochemical markers between primary and recurrent or metastatic breast cancer: A retrospective analysis of 107 cases

**DOI:** 10.1097/MD.0000000000020738

**Published:** 2020-06-19

**Authors:** Li Peng, Zhen Zhang, Dachun Zhao, Jialin Zhao, Feng Mao, Qiang Sun

**Affiliations:** a Department of Breast Surgery; b Department of Pathology, Peking Union Medical College Hospital, Chinese Academy of Medical Sciences and Peking Union Medical College, Beijing, P. R. China.

**Keywords:** discordance rate, estrogen receptor, human epidermal growth factor receptor-2, primary breast cancer, progesterone receptor, recurrent or metastatic lesions

## Abstract

There is a discordance in the immunohistochemical markers between primary breast cancer and recurrent or metastatic breast cancer. This study aimed to assess the recent trends and prognostic features in the treatment of recurrent or metastatic breast cancer

Overall, 107 patients were identified from January 2001 to August 2018 at the Peking Union Medical College Hospital, Beijing, and People's Republic of China to obtain a cohort of breast carcinoma patients who were confirmed to have recurrent or metastatic breast cancer by histopathology. We evaluated patient and tumor characteristics and examined the relationships between these factors and prognosis.

The estrogen receptor (ER), progesterone receptor (PR) and human epidermal growth factor receptor-2 (HER2) positivity, and Ki67 index in primary breast cancer were 63.6% (68/107), 58.9% (63/107), 19.8% (21/106) and 75.8% (75/99), respectively, while those in recurrent or metastatic lesions were 60.6% (65/107) (*P* = .672), 46.7% (50/107) (P = 0.013), 23.8% (25/105) (P = 0.482)and 83.5%(81/97)(P = 0.178), respectively. The discordance rate of HER2 expression was 10.6% (11/104), while that of PR expression was 23.3% (21/90). HER2 was the most stable biomarker. The discordance rates for luminal A and HER2 were as high as 100% and 25%, respectively, while the luminal B and triple negative values were as low as 8.3% and 5.3%, respectively.

ER and PR positivity and the Ki-67 index tended to increase due to recurrence or metastases; however, the discordance for PR and Ki-67 was high. PR is more variable than ER in the expression of primary and recurrent or metastatic breast cancer. The expression of HER2 receptor was the most stable and the discordance rate of triple negative breast cancer was the lowest. Therefore, although changes in biomarkers are due to recurrence or metastasis, pathological confirmation and exploration of markers are very important.

## 1 Introduction

Estrogen receptor (ER), progesterone receptor (PR), human epidermal growth factor receptor-2 (HER2), Ki-67 index, and p53 status are all important biomarkers associated with breast cancer. The biological characteristics of primary breast cancer and recurrence or metastasis are most important for guiding treatment and evaluating prognosis. Recent studies have shown that immunohistochemical markers may be differentially expressed in primary and recurrent or metastatic breast cancer, which can be attributable to the heterogeneity of breast cancer cell populations and the selective expression of receptors by cell cloning after treatment.^[1,2]^ The formulation of treatment regimens based on the expression of biomarkers has become the norm for primary breast cancer, but the guidelines for treatment of recurrent and metastatic breast cancer remain controversial; further, the status of biomarkers may change with the progression of the disease as the treatment proceeds.^[3,4]^ Therefore, it is important to study the characteristics of biomarkers in recurrent or metastatic lesions for individualized treatment of breast cancer. By observing the clinical and pathological data of patients with recurrent or metastatic breast cancer, this study analyzed the expression characteristics of ER, PR, HER2, Ki-67 index and p53 status in primary and recurrent or metastatic breast cancer, to provide evidence for the treatment and prognosis evaluation.

## 2 Patients and methods

### 2.1 Patients

A retrospective review of the data of 107 patients who presented with recurrent or metastatic breast cancer between January 2001 and August 2018 at the Peking Union Medical College Hospital was performed. Patients without a pathological diagnosis in our hospital were excluded from this data set. The medical records of patients with a pathologic diagnosis of recurrent or metastatic breast cancer were reviewed. We evaluated patient clinical characters, surgical procedures, histopathology and immunohistochemistry (IHC) characteristics, systemic treatment, and follow-up outcomes.

The Institutional Review Board of Peking Union Medical College Hospital (PUMCH) has exempted the study from IRB review as the study only involves the collection or study of the existing data or diagnostic specimens and these sources are publicly available or the information is recorded by the investigator in such a manner that subjects cannot be identified, directly or through identifiers linked to the subjects. The need for informed consent was waived owing to the retrospective design of the study.

### 2.2 Diagnostic approaches

Ultrasonography and CT/MRI were performed for diagnosis; however, histopathology is the gold standard method for diagnosis. Thus, the histopathology of primary breast cancer was evaluated based on the current WHO classification and patients were categorized based on their tumour-node-metastasis staging (TNM) classification. ER, PR, Ki-67 expression and p53 status were detected by IHC as described previously. HER2 expression was detected by IHC and HER2 was considered positive if the IHC test score was 3+. When the IHC test score was 2+, gene amplification status was determined using chromogenic in situ hybridization.

### 2.3 Adjuvant therapy and follow-up

Primary and recurrent or metastatic tumor status, number of lymph nodes removed/involved, histological grade, history of chemotherapy or radiotherapy, presence and medical care of recurrent or metastatic disease, and disease-free interval and overall survival (OS) were recorded.

### 2.4 Statistical analyses

Fisher exact and log rank tests were used to compare patient and tumor characteristics, and *P* < .05 was considered statistically significant. All statistical analyses were performed using the SSPS15.0 statistical software.

Need for informed consent was waived, and the research has been reported as per STROBE guidelines.

## 3 Results

We reviewed the pathology reports of 13891 women with breast cancer diagnosed between January 2001 and August 2018 at the Peking Union Medical College Hospital. The baseline information of all the patients enrolled in this study is presented in Table 1. Of these, a total of 107 patients were confirmed to have recurrent or metastatic tumor by histopathology. The mean age at presentation was 47.9 years (range: 23 to 88).

**Table 1:**
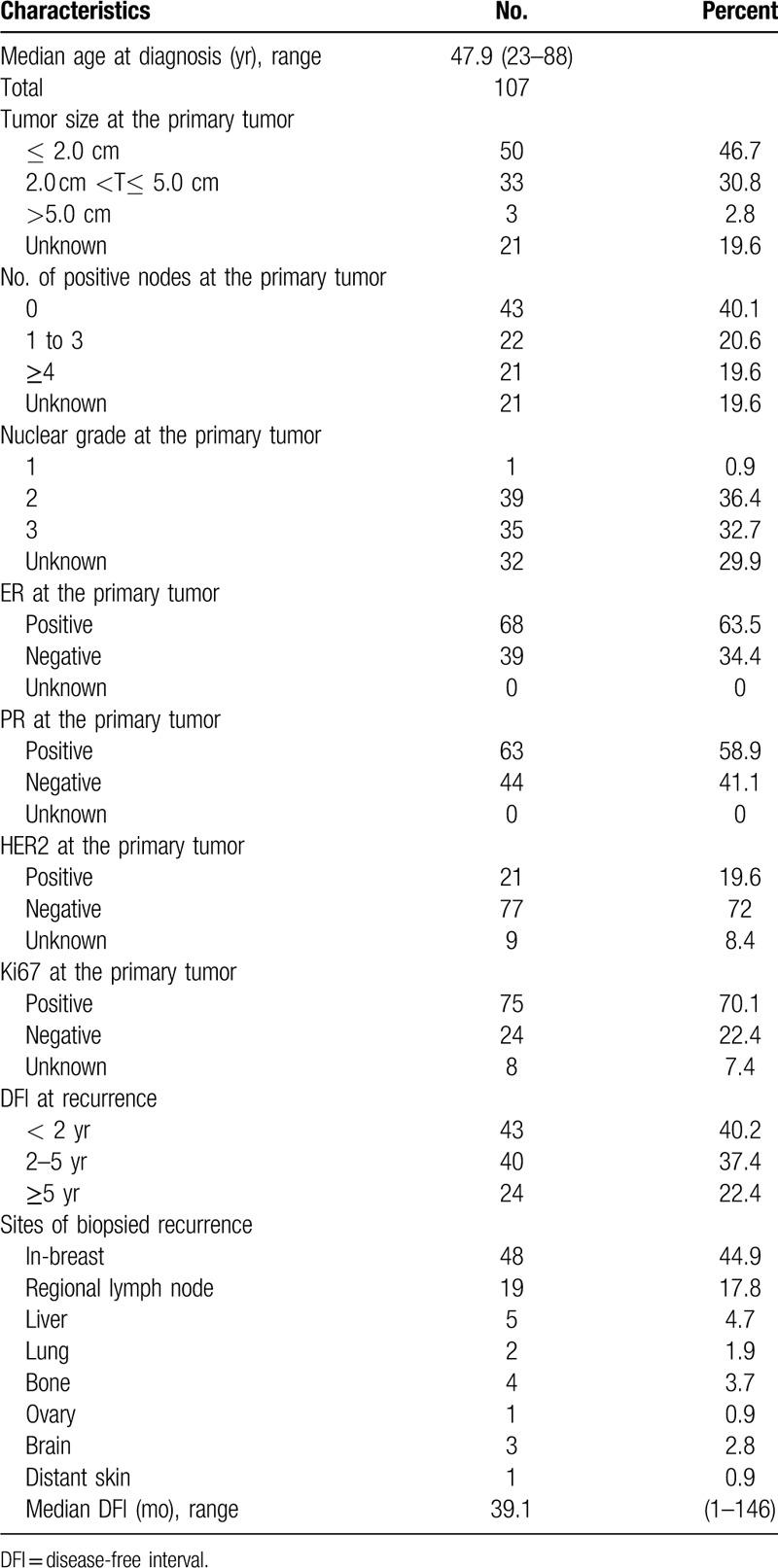
Characteristics of all patients with recurrent breast cancer in this study.

The median pathologic diameter was 2.4 cm (range: 0.5 to 12 cm).

The majority of patients (63/107, 58.9%) underwent mastectomy with axillary lymph node dissection and 8 patients underwent mastectomy with sentinel lymph node biopsy; 23 patients underwent lumpectomy with surgical axillary staging, while 12 patients who were older than 70 years underwent lumpectomy without surgical axillary staging. The last patient underwent tumor puncture for diagnosis. Overall, 86 patients (80.4%) underwent axillary node staging. Of these, 50% (43 of 86) had positive nodes and 12 patients did not have an axillary node staging because of advanced age. Forty-one patients had complete pathological staging performed. Median follow-up was 60.3 months (range 4 to 207), 5 patients died from distant metastasis and 1 patient died from a cerebrovascular accident.

In this study, chemotherapy protocols included epirubicin and cyclophosphamide with or followed by docetaxel; docetaxel and doxorubicin or epirubicin; cyclophosphamide and docetaxel; epirubicin and paclitaxel. Radiotherapy was recommended for only 44 of 107 patients; 19 patients received radiotherapy for lumpectomy while the remaining 25 patients received radiotherapy for large tumors or lymph node metastasis. Patients with hormone receptor-positive breast cancer were candidates for adjuvant endocrine therapy. Sixty-seven patients received adjuvant endocrine therapy. Eleven patients with HER2 receptor-positive disease received anti-HER2 therapy.

Changes in biological marker status (positive rates) between primary breast tumors and matched recurrent lesions are presented in Table 2. The positivity rates for ER, PR, HER2 expression, and Ki67 index in primary breast cancer were 63.6% (68/107), 58.9% (63/107), 19.8% (21/106), and 75.8% (75/99), respectively and for those in recurrent or metastatic lesions were 60.6% (65/107) (P = 0.672), 46.7% (50/107) (*P* = .013), 23.8% (25/105) (*P* = .482), and 83.5% (81/97) (*P* = .178), respectively. There was no significant difference between primary and recurrent or metastatic tumors.

**Table 2:**
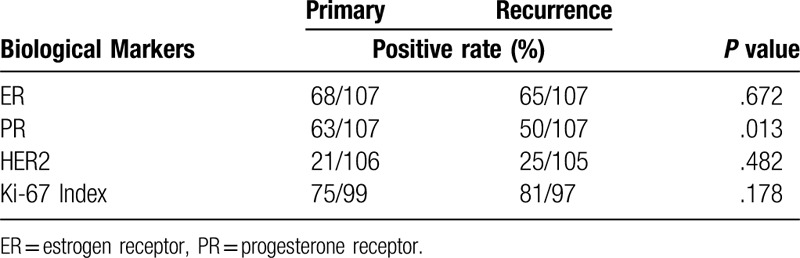
Changes in biological marker status (positive rates) between primary breast tumors and matched recurrent lesions.

Changes in biological marker status (category)between primary breast tumors and matched recurrent lesions are presented in Table 3. The expression of immunohistochemical markers was discordant between primary and recurrent or metastatic breast cancer. ER changed from positive to negative in 7 cases and from negative to positive in 4 cases. The discordance rate of ER expression was 10.3% (7/107). The ER status changed from positive to negative in 16 cases and from negative to positive in 3 cases. The discordance rate of PR expression was 17.8% (19/107). The HER2 status changed from positive to negative in 4 cases and from negative to positive in 7 cases. The discordance rate of HER2 expression was 10.6% (11/104). The Ki67 index changed from positive to negative in 7 cases and from negative to positive in 14 cases. The discordance rate of PR expression was 23.3% (21/90). HER2 was the most stable biomarker. In 87 patients with complete pathological information on the expression of ER, PR, HER2, and Ki67 index for primary tumor and recurrence or metastasis, there were 29 patients with 1 receptor shift, 7 patients with 2 receptor shifts and 51 patients without receptor shifts.

**Table 3:**
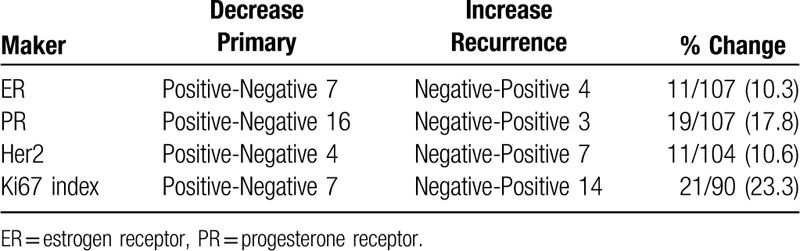
Changes in biological marker status (category)between primary breast tumors and matched recurrent lesions.

According to the new immunohistochemical results, 3 patents underwent surgical treatment only (104 patients underwent different surgical treatments), 42 patents received radiotherapy at the recurrence site, 65 received rescue chemotherapy, 55 received additional or modified endocrine therapy, 15 continued previous endocrine therapy, and 4 received bisphosphonate therapy for bone metastasis.

The mean follow-up time was (60.3 **±** 38.9, range 4–207 months). The mean disease-free interval time was (39.1 **±** 17.5, range 1–146 months). During the follow-up period, 6 patients died; 2 died of brain metastasis, 3 died of multiple organ metastasis and 1 died of cerebrovascular events.

Primary tumors and recurrent or metastatic subtypes are shown in Table 4. The discordance rates for luminal A and HER2 were as high as 100% and 25%, respectively, while the luminal B and triple negative (TN) values were as low as 8.3% and 5.3%, respectively. The molecular typing was based on the St. Gallen's Guide 2017.

**Table 4:**
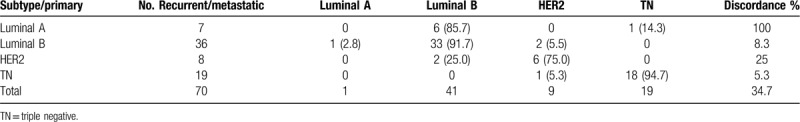
Changes in breast cancer subtype between primary breast tumors and matched recurrent lesions.

The 5- year OS rate was 82.0%. Univariate and multivariate analyses were performed on the OS -related factors for all patients (Table 5). Univariate factor analysis showed that grade and HER2 status of primary tumors, targeted therapy for both primary and recurrent tumors were important factors. Multivariate analyses showed that only rescue targeted therapy might be an independent factor affecting the results, but the significance of the results remains to be discussed due to the low mortality.

**Table 5:**
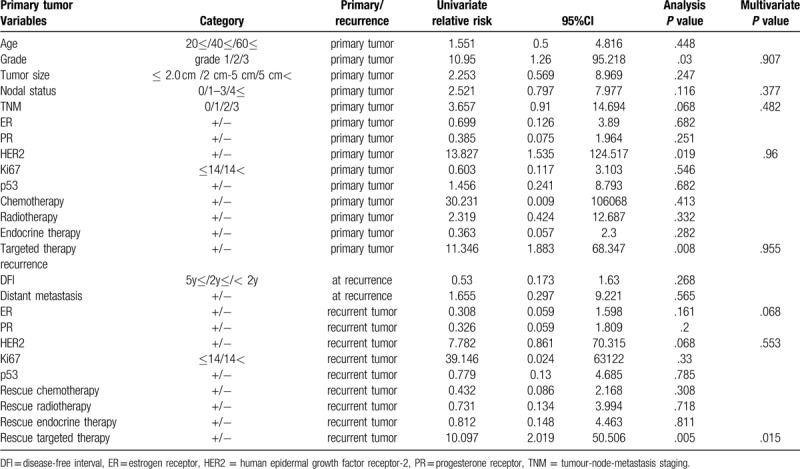
Uni- and multivariate analysis of factors for overall survival after recurrence.

## 4 Discussion

Breast cancer is a common malignant tumor and the second cause of cancer-related death in women. Recurrence and metastasis of breast cancer are the main causes of death. Discordance of ER, PR and HER2 status between the primary tumor and recurrent or metastatic tumor is well recognized; however, the mechanisms of discordance remain unknown. There are several potential explanations for the discordance in the reported literature, including variability in detection performance, tumor heterogeneity and biological evolution.^[5,6]^

Accuracy and reliability of immunohistochemical detection are based on factors such as tissue fixation, staining methods, selection of antigens and the pathologist's interpretation. Different periods, inspectors, and methods could affect the final results.^[7,8]^

Previous studies have shown that there are a small number of cancer cells that are prone to recurrence or metastasis in primary breast cancer. These cells may not be detectable at the initial diagnosis of primary breast cancer and may have different genetic characteristics.^[5,9]^

Therefore, the guidelines of the National Comprehensive Cancer Network (NCCN) clearly indicate that biopsy of recurrent and metastatic lesions is needed to identify the ER, PR and HER2 status of recurrent or metastatic lesions. However, some notable questions remain unanswered such as: How often does this phenomenon occur? What factors affect its occurrence? How do doctors deal with it according to its characteristics to improve survival rate?

This paper attempted to answer the first question in addition to the second and third. This paper also investigated the mechanism of receptor conversion in breast cancer and to assess if receptor conversion was reversible. Frequent changes in ER or PR status might be due to the selection or amplification of different tumor cell clones due to genetic or epigenetic mechanisms. During endocrine therapy, PR levels decreased significantly.^[10]^

Previous literature has reported that PR is more variable than ER in the expression of primary and recurrent or metastatic breast cancer.^[11]^ Similar changes were found in this study. For instance, the ER positivity rate decreased from 63.6% to 60.7% and the PR positivity rate decreased from 58.9% to 46.7%. Deletion of PR expression might play an important role in prognosis since ER-positive/PR-negative tumors are more invasive than ER-positive and PR-positive tumors. In the course of recurrence or metastasis of breast cancer, hormone receptor expression was partly absent and the deletion rate of PR in recurrence or metastasis was higher than that of ER, which may be related to the heterogeneity of tumors and previous endocrine therapy. Therefore, the absence of hormone receptors in tumor cells after recurrence or metastasis might lead to treatment failure in follow-up endocrine therapy.^[12]^

HER2 is 1 of the important genes related to breast cancer. High HER2 concordance between primary tumors and recurrent or metastatic tumor has been shown in many studies.^[13,14]^ In this study, the positive expression rate of HER2 in primary breast cancer was 19.8%, the positive expression rate in recurrent and metastatic breast cancer was 23.8%, and the expression discordance rate was 10.6% (11/104). In the discordant cases, HER2-positive metastases with negative primary tumors was more frequently seen than the opposite.^[15,16]^ In this study, HER2 changed from positive to negative in 4 cases and from negative to positive in 7 cases. Previous literature has shown that HER2 is differentially expressed in primary and recurrent or metastatic breast cancer^[17]^ and whether the patient was treated with trastuzumab at first diagnosis is a related factor.^[18]^ Anti-HER2 therapy has received great attention for use as adjuvant therapy, but whether anti-HER2 therapy should be added in rescue treatment needs further investigation.^[19,20]^ In those patients in whom HER2 changed from negative to positive, additional anti-HER2 therapy was needed. However, whether anti-HER2 therapy is necessary in patients whose HER2 changes from positive to negative still needs further confirmation. If the recurrence and metastasis are HER2-negative clones, whether anti HER2 therapy is effective remains controversial

Ki-67 index can accurately reflect the proliferative activity of tumor cells and is related to the development, metastasis, and prognosis of many kinds of tumors. In recent years, a gradually increased level of attention has been paid to this issue. The results showed that the expression of Ki-67 changed from high to low in 7 cases and from low to high in 14 cases. The expression inconsistency rate was 23.3% (21/90), which was similar to the rate of 22.6% reported by Nishimura et al.^[11]^

Breast cancer can be classified into 4 molecular subtypes based on the characteristics of receptor expression and there are obvious differences in the consistency of each sub-type. Higher expression discordance rates were identified with luminal A and B, while a lower expression discordance rate was found between HER2 and TN. The most consistent type of expression was the TN type and only 1 patient demonstrated discordance. This might be related to the high loss rate of PR and the increase in Ki-67 index, which led to the change from luminal A type to luminal B type. The expressions of HER2 and TN receptors were relatively stable, and their consistency is relatively high.^[21]^

In addition to surgical excision and radiotherapy as rescue treatment, some patients were treated with chemotherapy and endocrine therapy or had changes made to their endocrine treatment program. However, the long-term effect of this therapeutic switch has not been reported.^[22,23]^ Further randomized controlled trials in this setting no longer seem to be ethical and the optimal treatment choice for these patients is still a controversial topic.

To the best of our knowledge, this is so far 1 of the largest studies assessing discordance of immunohistochemical markers between primary and recurrent or metastatic breast cancer at 1 research center. But this study has some limitations. First, there might be methodological and selection biases due to the retrospective nature of this study. Because of the long-time span considered in the retrospective study and the differences in reagent use and standardization process in different periods, the differences might be minimized by repeated reviews, re-staining and interpretation of questionable pathological results based on the principle of being faithful to the original results at that time. Moreover, part of the patients’ medical information was lost and there might be biases due to data loss especially in the statistics of molecular types. In addition, the pathological information of metastases was difficult to obtain, and hence, some patients were excluded in this study. Although the author has objectively made statistical analysis of these data, there are still some minor regrets due to the data integrity. In fact, however, the study was conducted in a single institution to ensure that patients’ overall treatment policies were consistent. This study is meaningful because it is the largest study to compare the receptor status of those with primary breast cancer with both recurrence and metastasis at 1 clinical center in China.

Further investigation on how discordance arises is needed to improve our understanding of the topic and to perform clinical trials in the future. We hope to determine the risk of recurrence or progression by discordance and loss of progesterone expression whether is related to endocrine resistance mechanisms. Therefore, patients with progress or drug resistance should be included in the future study.

In conclusion, ER and PR decreased while Ki-67 tended to increase due to recurrence or metastases, but the discordance for PR and Ki-67 was high. PR is more variable than ER in primary and recurrent or metastatic breast cancer expression. The expression of HER2 receptor was the most stable and the discordance rate of TN breast cancer was the lowest. Therefore, although changes in biomarkers are due to recurrence or metastasis, pathological confirmation and exploration of markers are very important. Thus, there are still many unclear and uncertain issues in this field which need to be further explored.

## Acknowledgments

We are grateful to DR Yanna Zhang for her suggestions on the experimental design and statistics.

## Author contributions

Li Peng: Data curation, Investigation, Methodology, Resources, Project administration, Writing – original draft, Writing – review & editing.

Zhen Zhang: Formal analysis, Software.

Dachun Zhao: Methodology, Project administration.

Jialin Zhao: Project administration.

Feng Mao: Conceptualization, Funding acquisition, Investigation, Validation, Visualization, Supervision.

Qiang Sun: Conceptualization, Funding acquisition, Investigation, Validation, Visualization, Supervision.

Feng Mao, MD and Qiang Sun, MD have contributed equally to this work.
